# Study on the Development and Formation Specifics of Longissimus Dorsi Muscles in Ziwuling Black Goats

**DOI:** 10.3390/ani15223265

**Published:** 2025-11-11

**Authors:** Hailong Guo, Fuyue Shi, Lingrong Gu, Yanyan Wang, Yangyang Yue, Wei Huang, Yongqiang Yang, Panlong Sun, Wenyong Xue, Xiaoqiang Zhang, Xiaomei Zhu, Pengyang Shao, Yapeng He, Jianfeng Xu, Xiu Liu

**Affiliations:** 1 Animal Husbandry and Veterinary Research Institute of Gansu Province, Pingliang 744000, China; 2 Gansu Key Laboratory of Herbivorous Animal Science, College of Animal Science and Technology, Gansu Agricultural University, Lanzhou 730070, China; 3 Jingning County Animal Husbandry and Veterinary Center, Pingliang 743400, China; 4 Zhuanglang County Animal Husbandry and Veterinary Center, Pingliang 744600, China; 5 Huating City Animal Husbandry Science and Technology Development Service Center, Huating 744100, China; 6 Jingchuan County Animal Husbandry and Veterinary Center, Pingliang 744300, China; 7 Chongxin County Animal Husbandry and Veterinary Center, Pingliang 744200, China

**Keywords:** different ages, Ziwuling black goats, longissimus dorsi muscle, morphology, transcriptomics

## Abstract

To clarify the relationship between muscle development and meat quality in Ziwuling black goats, this study used the longissimus dorsi muscle of 6-month-old and 12-month-old goats as samples. With HE staining, fast–slow myofiber immunofluorescence double staining, and transcriptome sequencing, this study analyzed muscle structure, myofiber type transformation, and molecular regulation. Results showed that 6-month-olds had higher myofiber density and smaller diameter; 12-month-olds showed myofiber hypertrophy (larger diameter); immunofluorescence revealed more fast-twitch myofibers (Type II) at 6 months and increased slow-twitch ones (Type I) at 12 months. Transcriptome sequencing identified 387 differentially expressed genes (DEGs: 156 upregulated, 231 downregulated). GO analysis indicated that DEGs are involved in skeletal muscle growth, cAMP biosynthesis, etc.; KEGG analysis showed enrichment in arginine–proline metabolism and AMPK/MAPK signaling pathways (AMPK regulates fatty acid metabolism genes like *ACACB*/*CPT1A*; arginine–proline metabolism relates to muscle maturation). WGCNA clustered genes into nine modules (MEblue correlated with myofiber density/MAPK; MEgreen correlated negatively with diameter but positively with density, involving *PPARGC1A*/AMPK). In conclusion, protein nutrition at 6 months (promote myofiber proliferation) and regulating energy intake at 12 months (improve meat quality) are recommended, and 12 months is the optimal slaughter age.

## Simple Summary

This study aimed to clarify the relationship between muscle development and meat quality in Ziwuling black goats. We used the longissimus dorsi muscle of 6-month-old and 12-month-old goats as samples, and analyzed muscle structure, myofiber type transformation, and molecular regulation via HE staining, fast–slow myofiber immunofluorescence double staining, and transcriptome sequencing. Results showed that compared with 12-month-old goats, 6-month-old goats had higher myofiber density, smaller myofiber diameter, and more fast-twitch myofibers (Type II); in contrast, 12-month-old goats exhibited myofiber hypertrophy (larger diameter) and an increase in slow-twitch myofibers (Type I). Transcriptome sequencing identified 387 differentially expressed genes (DEGs: 156 upregulated, 231 downregulated). GO analysis indicated DEGs were involved in processes such as skeletal muscle growth and cAMP biosynthesis, while KEGG analysis showed DEGs were enriched in arginine–proline metabolism (related to muscle maturation) and AMPK/MAPK signaling pathways (AMPK regulates fatty acid metabolism genes like *ACACB*/*CPT1A*). Additionally, WGCNA clustered genes into nine modules, with key modules (e.g., MEblue, MEgreen) correlating with myofiber density, diameter, and MAPK/AMPK pathways. In conclusion, we recommend focusing on protein nutrition at 6 months (to promote myofiber proliferation) and regulating energy intake at 12 months (to improve meat quality); 12 months of age is determined as the optimal slaughter age for Ziwuling black goats.

## 1. Introduction

In the livestock and poultry breeding industry, meat quality and muscle development level are core factors that determine the industry’s economic benefits and ability to meet consumer demand, and they are also key indicators for measuring the superiority of livestock and poultry germplasm resources [1]. High-quality livestock and poultry meat not only require a tender texture and rich nutritional components but also rely on the reasonable development of muscle tissue. Specifically, the morphological structure of muscle fibers (such as diameter, density, and cross-sectional area), muscle fiber types, and their dynamic transformation process directly affect meat tenderness, the accumulation of flavor substances, and nutritional value. Moreover, differences in muscle development at different growth stages are an important basis for determining the appropriate slaughter period and optimizing breeding plans [2]. For local livestock and poultry breeds, in-depth analysis of their muscle development laws and molecular regulatory mechanisms can not only provide support for the scientific protection and efficient utilization of the breeds but also fill the gaps in the research field of muscle development for specific germplasm resources, thereby promoting the sustainable development of the local characteristic breeding industry.

The Ziwuling black goat is a traditional livestock breed in Western China, mainly distributed in the producing areas around the Ziwuling Mountains. It serves as a crucial germplasm resource and an industrial pillar for the local mutton sheep industry [3]. This breed is prominently characterized by its black coat. It not only has adaptive advantages such as good roughage tolerance and strong stress resistance but also stands out for its excellent trait of tender and delicious meat. Its meat quality is significantly superior to that of other common goat breeds, making it a crucial germplasm resource and industrial pillar for the development of the mutton sheep industry in the producing areas [4]. The longissimus dorsi muscle, as the core muscle group of its body, has a developmental status that serves as a key indicator for evaluating the meat production performance and meat quality of meat-producing animals. It is directly related to the muscle fiber structure, the accumulation of nutritional components, and, ultimately, the economic value of the meat product [5]. Muscle development is a dynamic process that involves cell proliferation and tissue differentiation, with significant differences in its morphology and function across different monthly age stages [6,7]. At 6 months of age, a critical transition stage from infancy to adolescence, muscle tissue proliferates rapidly, accompanied by the initial differentiation of fiber types. At 12 months of age, the animals are close to sexual maturity, and muscle development tends to stabilize; this is an important period for the formation of meat quality traits. Indicators such as muscle fiber diameter, density, and cross-sectional area are important bases for evaluating muscle quality [8], Hematoxylin and Eosin (HE) staining technique can clearly display the morphological structure and arrangement characteristics of muscle fibers [9]. In addition, the composition and transformation of muscle fiber types are also key factors affecting meat quality. Skeletal muscle is mainly composed of fast-twitch muscle fibers (Type II) and slow-twitch muscle fibers (Type I), and the double immunofluorescence technique enables accurate identification and analysis of these fibers [10,11]. Studies have shown that muscle fiber characteristics are regulated by multiple factors such as age [12,13], diet composition [14], and rearing environment [15]. At the molecular mechanism level, transcriptomics technology has been widely applied in studies related to muscle development. For example, Pan et al. [16] identified 231 differentially expressed genes associated with meat quality and muscle development by comparing two goat breeds. Cao et al. [17] analyzed the dynamic changes in skeletal muscle development of Hu sheep from 3 days after birth to 12 months of age, identified 6865 differentially expressed genes, and suggested that genes such as ARID5B, MYOG, and ENO1 are related to muscle hypertrophy, while genes like NR1D1 and FADS1 are associated with muscle fiber type transformation. Other studies have also been conducted focusing on different breeds [18], ages [19], and developmental stages [20], gradually uncovering the molecular regulatory network of muscle development.

At present, in-depth research on the systematic developmental characteristics of the longissimus dorsi muscle during key growth and development stages is still lacking for the Ziwuling black goat, a specific local breed. Therefore, this study took the longissimus dorsi muscle of 6-month-old and 12-month-old Ziwuling black goats as the research subjects. It comprehensively used HE staining to observe tissue morphology, double immunofluorescence staining to analyze the transformation law of fast-twitch and slow-twitch muscle fiber types, and transcriptome sequencing to screen key differentially expressed genes, so as to systematically explain the developmental characteristics and molecular regulatory mechanisms of the longissimus dorsi muscle at these two important monthly age stages. The research results aim to provide a theoretical basis for the precise nutritional regulation and determination of the appropriate slaughter period of Ziwuling black goats. At the same time, the research results further enrich the basic data on muscle development of local goat germplasm resources and provide support for their scientific protection and efficient utilization.

## 2. Materials and Methods

### 2.1. Experimental Location and Time

Healthy Ziwuling black goats at 6 and 12 months of age were selected as experimental subjects from the same herdsman’s flock in Huan County, Qingyang City, Gansu Province. Each age group consisted of 6 goats: the 6-month-old group (designated as B6) and the 12-month-old group (designated as B12). All goats were slaughtered to collect longissimus dorsi muscle samples, with ruminant sample collection conducted concurrently in strict accordance with the experimental protocol. Slaughter was performed via jugular vein exsanguination on the early morning of the day following sample preparation. Prior to slaughter, the experimental goats were subjected to a 12 h fasting period and a 2 h water deprivation period to standardize physiological conditions. Longissimus dorsi muscle samples were collected within 30 min post-slaughter to ensure tissue freshness. Notably, this slaughter experiment was reviewed and approved by the Animal Ethics Committee of Gansu Agricultural University, with the official approval number GAU-LC-2020-27.

### 2.2. Sample Collection

After slaughter, the longissimus dorsi muscle was taken. One part was fixed with 4% paraformaldehyde, which was used for histological and morphological analysis and immunofluorescence double staining; the other part was placed in 1.5 mL cryopreservation tubes (three tubes per goat) and immediately stored in a pre-prepared liquid nitrogen tank for freezing. After being transported back to the laboratory, the samples were stored at −80 °C for subsequent RNA-Seq analysis.

### 2.3. Histo- and Morphlolgical Analysis

A 1 cm^2^ sample of longissimus dorsi muscle tissue was fixed in 4% paraformaldehyde solution. After 24 h of fixation, the tissue samples underwent a standard series of histological procedures, including dehydration, clearing, paraffin infiltration, embedding, sectioning, and staining, to prepare paraffin-embedded tissue sections. Hematoxylin–Eosin (HE) staining was performed for morphological observation: cell nuclei were stained blue–purple, while cytoplasm was stained pink. For each longissimus dorsi muscle sample, three non-adjacent HE-stained sections were selected, and digital images were captured using a microscope equipped with an imaging system.

### 2.4. Immunofluorescence Staining

#### 2.4.1. Deparaffinization of Paraffin Sections to Water

Sections were sequentially immersed in environment-friendly dewaxing solution I, II, and II for 10 min each, followed by anhydrous ethanol I, II, and III for 5 min each. Finally, sections were rinsed with distilled water.

#### 2.4.2. Antigen Retrieval

Sections were placed in EDTA (pH 8.0) retrieval buffer and treated with a microwave oven under medium power for 10 min→power off for 5 min→medium-low power for 5 min→power off for 2 min→medium-low power for 5 min (to prevent buffer evaporation and section drying). After natural cooling, sections were transferred to PBS (pH 7.4) and washed 3 times on a decolorizing shaker, 5 min each time.

#### 2.4.3. Circle Drawing and Serum Blocking

Sections were spun dry, and a histochemical pen was used to draw a circle around the tissue. Blocking solution was added (10% donkey serum for primary antibodies of goat origin, 3% BSA for primary antibodies of other origins), and blocking was performed for 30 min.

#### 2.4.4. Primary and Secondary Antibody Incubation

Primary antibody incubation: Two primary antibodies were mixed and prepared (rabbit-derived fast antibody, 1:300; mouse-derived slow antibody, 1:500), added dropwise to the sections, then placed flat in a humid chamber and incubated overnight at 4 °C. Secondary antibody incubation: Sections were transferred to PBS (pH 7.4) and washed 3 times on a decolorizing shaker (5 min each time). Corresponding secondary antibodies were added dropwise (CY3-labeled goat anti-rabbit IgG, 1:300; Alexa Fluor 488-labeled goat anti-mouse IgG, 1:400) and incubated at room temperature for 50 min in the dark.

#### 2.4.5. DAPI Counterstaining and Mounting

After sections were washed 3 times with PBS (pH 7.4) on a decolorizing shaker (5 min each time), DAPI staining solution was added dropwise, and incubation was carried out at room temperature for 10 min in the dark. Sections were then washed 3 times with PBS (5 min each time), treated with auto-fluorescence quencher Solution B for 5 min, rinsed with running water for 10 min, and finally mounted with anti-fluorescence quenching mounting medium.

#### 2.4.6. Image Acquisition

Tissue sections were loaded onto a PANNORAMIC whole-slide scanner (3DHISTECH, Budapest, Hungary). The sections moved gradually under the scanner’s lens, imaging while moving to scan all tissue information on the sections into a folder containing all tissue information from the sections. The folder could be opened with CaseViewer 2.4 software for observation at any magnification from 1× to 400×. Muscle tissue was selected for imaging at 400× magnification, with the tissue filling the entire field of view as much as possible and with consistent background light for each photo. After imaging, Image-Pro Plus 6.0 (Table 1) analysis software was used to measure the diameter of muscle fibers at 5 locations under 400× magnification and calculate the average value; measure the total area of muscle fibers in 3 fields of view per section and count the number of muscle fibers in the fields of view; count the number of slow-twitch muscles, fast-twitch muscles, and total muscle fibers in 3 fields of view using immunofluorescence, and calculate the slow-twitch muscle ratio (%) = (number of slow-twitch muscles/total number of muscle fibers) × 100, and fast-twitch muscle ratio (%) = (number of fast-twitch muscles/total number of muscle fibers) × 100. (Details of the main instruments, reagents, primary antibodies, secondary antibodies, and corresponding antigen retrieval conditions used in this experiment are shown in Supplementary Tables S1–S3.)

### 2.5. Transcriptome Sequencing Analysis of Goat Longissimus Dorsi Muscle

#### 2.5.1. RNA Extraction, Library Construction, and Sequencing

Total RNA was extracted from longissimus dorsi muscle tissue using the Trizol reagent kit (Invitrogen, Carlsbad, CA, USA). All extraction procedures were performed strictly according to the kit instructions and conducted on an ultra-clean bench. A micro-spectrophotometer (Therm Nano Drop-2000, Waltham, MA, USA) was used to detect the concentration and purity of the extracted RNA, recording the RNA concentration (ng/μL) and purity (260 nm/280 nm ratio of 1.8–2.1). The VAHTS Universal V6 RNA-seq Library Prep Kit for Illumina^®^ (NR604-02, Vazyme, Nanjing, China) was used to construct cDNA libraries of the samples, and the VAHTSTM DNA Clean Beads kit (N411-03, Vazyme, Nanjing, China) was used to purify the products. The constructed cDNA libraries were sequenced on the Illumina NovaSeq 6000 system (San Diego, CA, USA).

#### 2.5.2. Alignment with Reference Genome

The raw image data obtained from Illumina platform sequencing were converted into raw sequencing reads (Raw Reads) through base calling. Quality control was performed on Raw Reads to obtain high-quality sequences (Clean Reads). The Clean Data were aligned with the reference genome (Oar_rambouillet_v1.0.Ovis_aries) using HISAT to obtain Mapped Data. StringTie (v3.0.1) was used to assemble the aligned reads, and FPKM was adopted as the indicator to measure the expression levels of transcripts or genes.

#### 2.5.3. Functional Annotation Analysis of Differentially Expressed Genes

DESeq2 (v1.49.4) was used for differential gene expression analysis, with the screening criteria of FC ≥ 2 and FDR < 0.01 for identifying differentially expressed genes. GO and KEGG functional enrichment analyses of the differential genes were performed using GOseq (v1.61.1).

#### 2.5.4. Validation by Real-Time Quantitative PCR (RT-qPCR)

The reliability of the transcriptome sequencing data was verified by RT-qPCR. Eight genes were randomly selected from the screened differentially expressed genes, and their relative expression levels were determined. Information on the gene primers is shown in Table 2.

### 2.6. Data Analysis

The experimental data were initially organized using Excel 2016, then statistically analyzed via independent samples *t*-test in SPSS 24.0 software. The relative expression levels of genes were analyzed using the 2^−∆∆CT^ [21] method. For measurements of HE-stained sections and immunofluorescence double-stained sections, Image-Pro Plus 6.0 was used with “millimeter (mm)” as the standard unit uniformly. The results were visualized using Prism software and expressed as “mean ± standard deviation”. The value of *p* < 0.05 was considered statistically significant (Table 2).

## 3. Results

### 3.1. Analysis of Morphological Characteristics of Longissimus Dorsi Muscle

**Figure 1:**
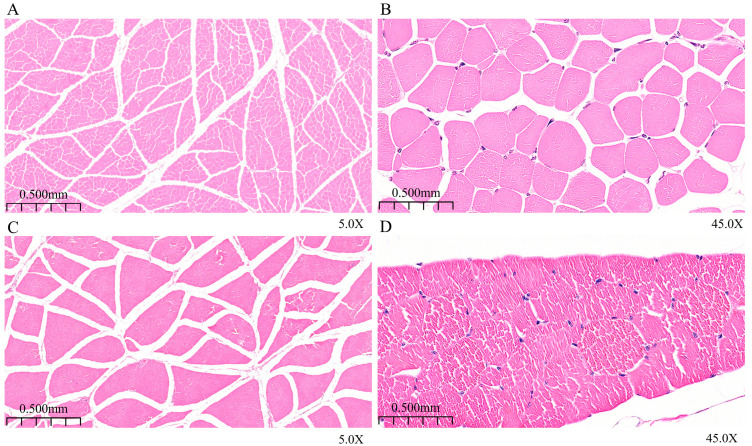
HE-stained histomorphological sections of the longissimus dorsi muscle. (**A**,**B**): HE-stained sections of the longissimus dorsi muscle from 6-month-old specimens; (**C**,**D**): HE-stained sections of the longissimus dorsi muscle from 12-month-old specimens.

**Figure 2:**
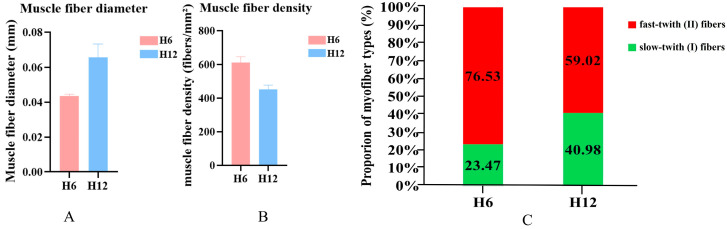
Measurement results of HE sections and immunofluorescence double staining. (**A**): Measurement results of muscle fiber diameter in longissimus dorsi muscle at 6 months and 12 months of age; (**B**): measurement results of muscle fiber density in longissimus dorsi muscle at 6 months and 12 months of age; (**C**): measurement results of fast-twitch muscle fibers and slow-twitch muscle fibers in longissimus dorsi muscle at 6 months and 12 months of age.

By observing and analyzing HE-stained sections of the longissimus dorsi muscle from 6-month-old and 12-month-old Ziwuling black goats (Figure 1 and Figure 2A,B), it was found that the 6-month-old group had relatively higher muscle fiber density and smaller fiber diameter, while the 12-month-old group exhibited increased muscle fiber diameter and decreased fiber density.

### 3.2. Analysis of Immunofluorescence Staining Characteristics of Longissimus Dorsi Muscle

**Figure 3:**
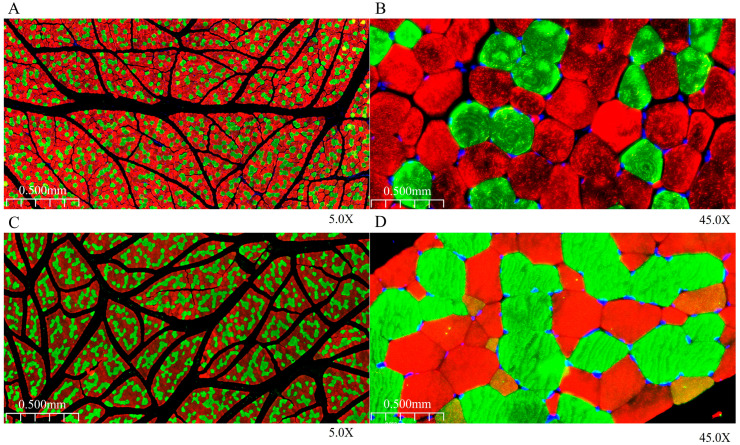
Immunofluorescence staining of the longissimus dorsi muscle. (**A**,**B**): immunofluorescence staining of the longissimus dorsi muscle from 6-month-old specimens; (**C**,**D**): immunofluorescence staining of the longissimus dorsi muscle from 12-month-old specimens. Note: immunofluorescence staining showing slow-twitch muscle fibers (Type I, green), fast-twitch muscle fibers (Type II, red), and myocyte nuclei (blue).

By performing immunofluorescence staining and analysis on the longissimus dorsi muscle (Figure 2C and Figure 3), it was found that the muscle fibers of the 6-month-old longissimus dorsi muscle were dominated by fast-twitch muscle fibers (Type II). In contrast, the proportion of slow-twitch muscle fibers (Type I) in the 12-month-old group gradually increased. Specifically, the proportion of Type I fibers was 23.47% in the 6-month-old group and increased to 40.98% in the 12-month-old group, while the proportion of Type II fibers was 76.53% in the 6-month-old group and decreased to 59.02% in the 12-month-old group.

### 3.3. Quality Assessment of Longissimus Dorsi Muscle RNA-Seq Data

By analyzing the base composition and quality distribution of the sequencing data (Supplementary Figure S1), it was found that the base distribution (A, T, C, G) was balanced across all 12 samples, and the Q30 values were all greater than 94.21% (Supplementary Table S1). These results indicate that the sequencing data are of high quality and meet the requirements of subsequent experiments.

### 3.4. Alignment of Longissimus Dorsi Muscle RNA-Seq Data with Reference Genome

The HISAT2 software was used for rapid and accurate alignment of Clean Reads with the goat reference genome (Ovis_aries.Oar_rambouillet_v1.0) (Supplementary Table S2) to obtain the positioning information of reads on the reference genome. Subsequently, StringTie was used to assemble the aligned reads, and the transcriptome was reconstructed for subsequent analysis.

### 3.5. Identification of Differentially Expressed Genes

**Figure 4:**
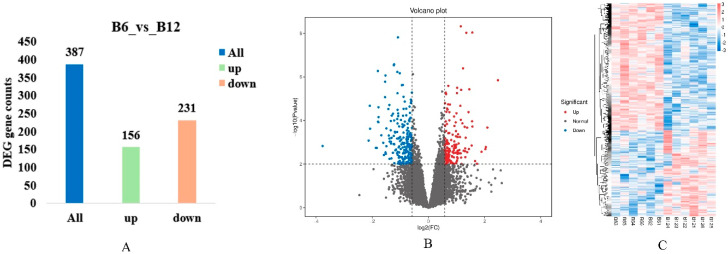
Analysis of differentially expressed genes in Longdong black goats. (**A**): Bar chart of differentially expressed genes; (**B**): volcano plot of differentially expressed genes; (**C**): heatmap of differentially expressed genes.

Using |Log FC| ≥ 2 and FDR < 0.01 as the screening criteria, a total of 387 differentially expressed genes (DEGs) were identified in the B6_vs_B12 comparison group, including 156 upregulated genes and 231 downregulated genes. A heatmap was generated based on the expression levels of the screened differentially expressed genes. The results showed that the variation in the expression levels of differentially expressed genes was small within groups but large between groups (Figure 4).

**Figure 5:**
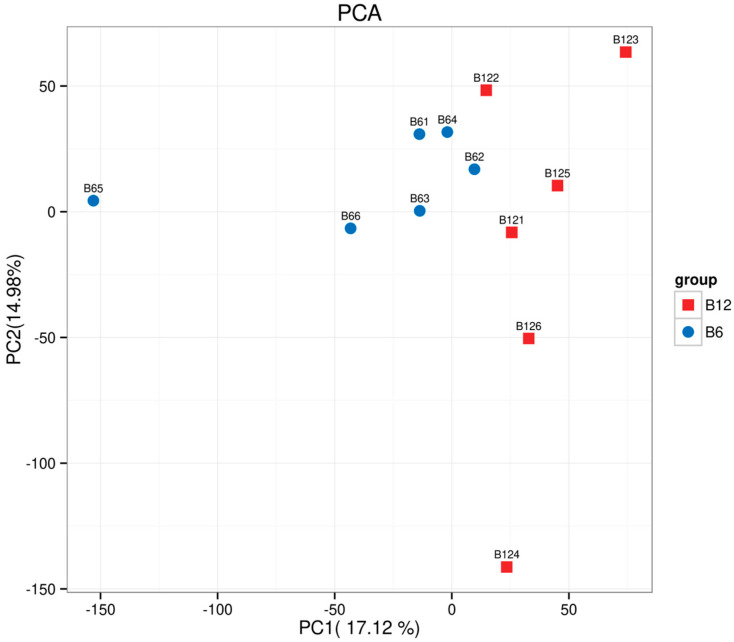
Transcriptome sequencing analysis of the longissimus dorsi muscle. Note: PCA showed that samples from the 6-month-old and 12-month-old groups were clearly clustered, and the differences between groups were extremely significant (*p* = 0.004) as verified by ANOVA (Supplementary Table S6).

Transcriptome analysis was performed on Ziwuling black goats of different ages, yielding a total of 70.17 Gb of Clean Data, with each sample generating at least 5.73 Gb of Clean Data. Principal Component Analysis (PCA) showed distinct intra-group clustering and significant inter-group differences (Figure 5 and Supplementary Table S6).

### 3.6. Functional Annotation of Differentially Expressed Genes

#### 3.6.1. GO Enrichment Analysis

**Figure 6:**
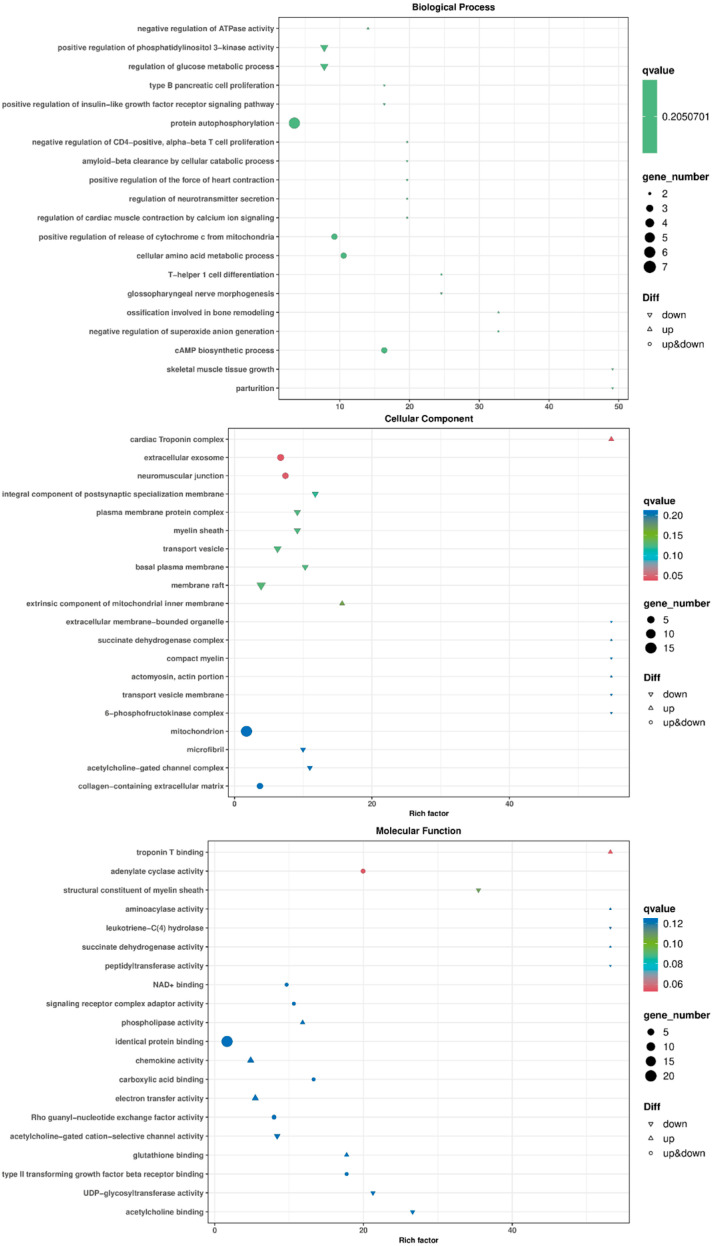
GO classification map of differentially expressed genes in Ziwuling black goats of different ages.

To clarify the functions of DEGs in Ziwuling black goats during meat quality regulation, GO functional annotation was performed on the differentially expressed genes through biological process (GO-BP), cellular component (GO-CC), and molecular function (GO-MF). Through the analysis of the annotation results, it was found that in BP, the differentially expressed genes mainly played roles in the regulation of skeletal muscle tissue growth (GO:0048630), cAMP biosynthetic process (GO:0006171), negative regulation of superoxide anion generation (GO:0032929), cellular amino acid metabolic process (GO:0006520), and the regulation of glucose metabolic process (GO:0010906). In CC, the differentially expressed genes were mainly involved in biological processes such as collagen-containing extracellular matrix (GO:0062023), actomyosin, actin portion (GO:0042643), and mitochondrion (GO:0005739). In MF, they mainly functioned in processes such as glutathione binding (GO:0043295), succinate dehydrogenase activity (GO:0000104), NAD+ binding (0070403), and electron transfer activity (GO:0009055) (Figure 6).

#### 3.6.2. KEGG Signaling Pathway Enrichment Analysis

**Figure 7:**
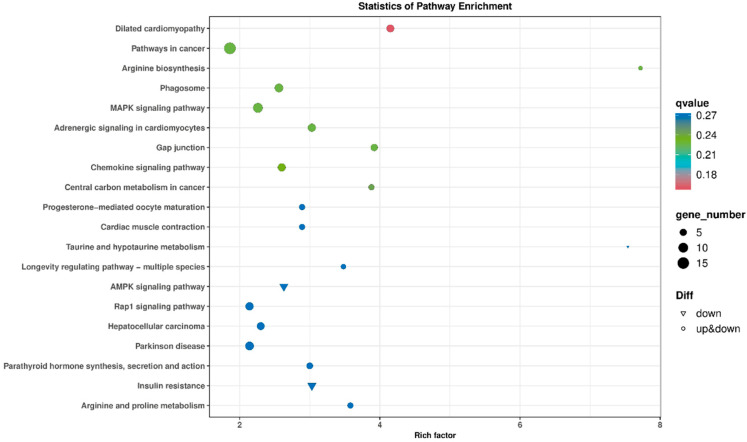
KEGG pathway annotation of differentially expressed genes in Ziwuling black goats of different ages.

KEGG functional enrichment analysis was conducted on the DEGs of Ziwuling black goats at different ages. The results showed that these DEGs were mainly enriched in pathways including arginine and proline metabolism (ko00330), AMPK signaling pathway (ko04152), gap junction (ko04540), MAPK signaling pathway (ko04010), and Chemokine signaling pathway (ko04062). Among them, the downregulated genes were primarily enriched in pathways such as AMPK signaling pathway (ko04152) and insulin resistance (ko04911) (Figure 7 and Table 3).

### 3.7. RT-qPCR Validation

**Figure 8:**
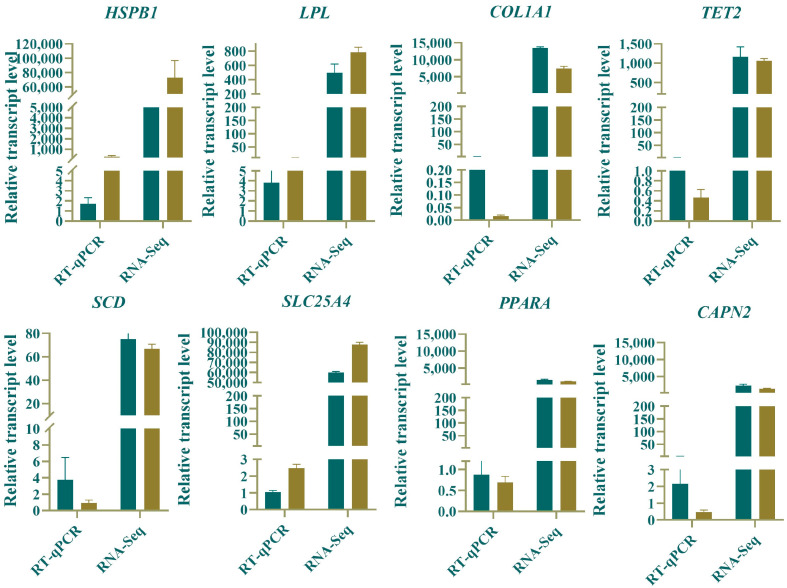
RT-qPCR and RNA-Seq validation. Note: For both RT-qPCR and RNA-Seq, the expression level on the left represents that of 6-month-old specimens, and the one on the right represents that of 12-month-old specimens.

Eight genes were randomly selected from the differentially expressed genes in the longissimus dorsi muscle of Ziwuling black goats at different monthly ages, and their expression levels were validated using the qPCR method. As shown in Figure 8, the expression trends detected by RT-qPCR were consistent with the results of RNA-Seq analysis, indicating that the RNA-Seq data had high reliability.

### 3.8. Weighted Gene Co-Expression Network Analysis (WGCNA)

**Figure 9:**
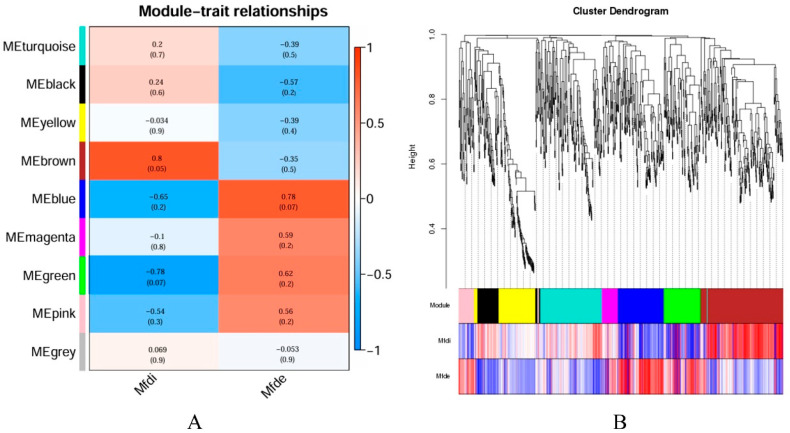
Weighted gene co-expression network analysis results. (**A**) Module-trait relationships; (**B**) Cluster dendrogram.

Based on RNA transcriptome data, weighted gene co-expression network analysis (WGCNA) was performed, which revealed that the expressed genes in the rumen of Ziwuling black goats at different months of age were clustered into nine gene modules. These gene modules exhibited varying degrees of correlation with rumen muscle fiber diameter and muscle fiber density, among which the modules with stronger correlations included MEbrown, MEblue, MEgreen, Megrey, and Mepink. Specifically, MEbrown was significantly positively correlated with muscle fiber diameter (*p* < 0.05); MEblue was significantly positively correlated with muscle fiber density (*p* < 0.05), and the co-expressed host genes (*GADD45A*, *FGFR3*) in these modules were associated with functions like the “MAPK signaling pathway”; MEgreen was significantly negatively correlated with muscle fiber diameter (*p* < 0.05) while being significantly positively correlated with muscle fiber density (*p* < 0.05), and the co-expressed host gene *PPARGC1A* in this module was associated with the function of the “AMPK signaling pathway” (Figure 9).

## 4. Discussion

Morphological parameters of muscle fibers are key indicators reflecting muscle growth status and meat quality characteristics [22]. In this study, Ziwuling black goats at 6 months of age are in the transition stage from the juvenile to the young adult period, with muscle tissue mainly undergoing rapid proliferation. This is manifested by a higher muscle fiber density and smaller diameter. This change pattern is basically consistent with the muscle development process of meat-producing animals such as pigs [23] and sheep [24], indicating that the transition of muscle fibers from “proliferation-dominated” to “hypertrophy-dominated” is a common feature of mammalian muscle growth. The composition of muscle fiber types is closely related to muscle metabolic characteristics, contractile function, and meat flavor formation [25]. In this study, the proportion of fast-twitch muscle fibers (Type II) in the muscles of 6-month-old goats is relatively high, which helps to meet the demand for rapid energy supply during the active movement of animals in the juvenile stage. In contrast, the proportion of slow-twitch muscle fibers (Type I) increases significantly at 12 months of age, reflecting a gradual shift in muscle metabolism from glycolysis-dominated to oxidative phosphorylation-dominated. Slow-twitch muscle fibers are rich in mitochondria and myoglobin, which contribute to improving muscle water-holding capacity and promoting the accumulation of flavor substances [26]. This may be the structural basis for the richer meat flavor of Ziwuling black goats at 12 months of age. Therefore, from the perspective of muscle fiber morphology and type transformation, the period of 6–12 months of age is a critical window for the formation of muscle texture characteristics in Ziwuling black goats.

Ziwuling black goat is an important local goat breed in China, and its muscle development process has breed-specific characteristics. The longissimus dorsi muscle, a typical site for muscle growth and meat production efficiency, is often used in studies on related physiological mechanisms [27]. In this study, transcriptome sequencing was performed on the longissimus dorsi muscle of Ziwuling black goats at 6 and 12 months of age, and a total of 387 DEGs were identified, including 156 upregulated genes and 231 downregulated genes. This indicates that there are significant differences in the molecular regulatory mechanisms of muscle development between the two age stages, and these genes may regulate the transformation of muscle fiber structure and metabolic function by participating in specific biological processes and signaling pathways.

GO functional enrichment analysis showed that in the biological process category, DEGs were significantly enriched in pathways such as skeletal muscle tissue growth, cAMP biosynthesis, and glucose metabolism. Among them, the expression dynamics of genes related to skeletal muscle tissue growth are directly associated with the morphological transition of muscle fiber proliferation and hypertrophy [28]. As a key second messenger, the biosynthesis process of cAMP participates in the regulation of myocyte proliferation and differentiation by regulating the PKA signaling pathway [29]. Fast-twitch muscle fibers at 6 months of age mainly rely on glycolysis, while slow-twitch muscle fibers at 12 months of age are more dependent on fatty acid oxidation. This metabolic shift may be driven by the expression regulation of nuclear receptor genes such as *PPARA*. In terms of cellular components, DEGs are significantly enriched in structures such as collagen-containing extracellular matrix and mitochondria. The upregulated expression of mitochondria-related genes provides support for the oxidative metabolic capacity of slow-twitch muscle fibers [30], which is consistent with the results of muscle fiber type transformation observed by immunofluorescence. The enrichment of extracellular matrix-related genes is closely related to the development and maturation of muscle connective tissue. In addition, the enrichment of the MAPK signaling pathway suggests that it participates in the signal transduction process of cell growth and differentiation during the rapid muscle proliferation stage at 6 months of age, further explaining the differences in muscle development between different age groups at the molecular level [31].

KEGG pathway enrichment analysis revealed the core molecular regulatory network underlying the developmental differences in the longissimus dorsi muscle between 6-month-old and 12-month-old Ziwuling black goats. These pathways synergistically participate in regulating muscle growth, metabolic transformation, and functional maturation [32]. Arginine, a conditionally essential amino acid [33], not only serves as a precursor for protein synthesis but also regulates vasodilation through the NO synthesis pathway, thereby influencing muscle blood flow and nutrient supply [34]; proline, the main component of collagen, has a metabolic level that directly affects the synthesis and remodeling of connective tissue [35,36]. In this study, the differential expressions of *NOS1* and *SMOX* genes in this pathway suggest that 12-month-old goats may enhance arginine metabolism to improve muscle blood perfusion and promote the conversion of proline to collagen. This is consistent with the observed phenotype of connective tissue accumulation, indicating that this pathway is an important basis for supporting muscle structural maturation. The AMPK signaling pathway, a core pathway for energy sensing and metabolic regulation, plays a key role in maintaining muscle energy homeostasis [37,38]. By regulating the expression of genes related to fatty acid oxidation (e.g., CPT1A) and fat synthesis (e.g., ACACB), this pathway coordinates the balance between energy supply and demand [39,40]. The downregulation of *ACACB* and *CPT1A* in this study may reflect that 6-month-old muscle relies on glycolysis and fat synthesis to meet the energy needs of rapid proliferation, while 12-month-old muscle shifts to a fatty acid oxidation-dominated metabolic pattern to support the oxidative metabolic characteristics of slow-twitch muscle fibers. This demonstrates the adaptive regulation of the AMPK pathway in response to the energy metabolism demands of different developmental stages. The enrichment of the gap junction pathway suggests that there are developmental differences in intermyocyte communication. This pathway mediates the synchronization of intercellular signals and the exchange of metabolites through connexins, thereby affecting the coordinated contraction ability of muscles [41]. The differential expression of *ADCY6* and *ADCY7* genes may be involved in intercellular signal transmission by regulating cAMP levels [42]. The function of this pathway tends to be improved in 12-month-old individuals, which helps enhance muscle contraction efficiency and aligns with the physiological characteristic of stable motor ability during sexual maturity. The MAPK signaling pathway is involved in the temporal regulation of muscle cell proliferation and differentiation [43]. Through cascade reactions, this pathway transduces extracellular signals [44] and affects processes such as cell proliferation, differentiation, and apoptosis [45]. In 6-month-old muscles, this pathway may be activated to promote satellite cell proliferation and muscle fiber formation; however, during the stable developmental stage at 12 months of age, its activity may be downregulated accordingly to avoid excessive proliferation. The enrichment of the chemokine signaling pathway suggests that immune and inflammatory responses play a potential role in muscle development [46,47]. Chemokines recruit immune cells to participate in tissue repair and regeneration [48], and their expression changes in 12-month-old muscles may be related to local immune regulation induced during muscle fiber type transformation and connective tissue remodeling [49]. The differential expression of chemokine-related genes in 12-month-old muscle may be associated with local immune responses induced by tissue remodeling (e.g., collagen cross-linking and myofiber type transformation) during muscle maturation [50], providing a microenvironmental support for the stability of muscle structure [51]. Further analysis using weighted gene co-expression network analysis (WGCNA) identified nine gene modules associated with muscle fiber diameter and density. Among these, the MEbrown module showed a significant positive correlation with muscle fiber diameter, and the co-expressed gene *GADD45A* within this module is associated with the MAPK signaling pathway. Studies have shown that *GADD45A* is involved in a variety of cellular processes and can regulate the p38MAPK pathway [52]. In goats, the relevant genes can affect myocyte differentiation by activating the p38MAPK [53]. The co-expressed gene *PPARGC1A* in the MEgreen module is associated with the AMPK signaling pathway. *PPARGC1A* plays a core role in energy metabolism and cell function regulation and may affect metabolic status as well as cell proliferation and differentiation through the AMPK pathway, thereby regulating muscle fiber morphology [54].

To sum up, the 6-month-old and 12-month-old stages are critical transition periods for the development of the longissimus dorsi muscle in Ziwuling black goats, during which significant changes occur in muscle fiber morphology, metabolic functions, and molecular regulatory mechanisms. The results of this study suggest that during the 6-month-old stage, emphasis should be placed on protein nutrition to promote muscle fiber proliferation, while during the 12-month-old stage, rational regulation of energy intake is required to optimize meat quality. Based on the characteristics of muscle fiber diameter, density, and the accumulation of related substances, it is recommended that the appropriate slaughter age for Ziwuling black goats is around 12 months old. The findings of this study not only provide a theoretical basis for further understanding of the muscle development mechanism of local goats but also lay a scientific foundation for the efficient utilization of the germplasm resources of Ziwuling black goats.

## 5. Conclusions

This study systematically analyzed the developmental characteristics and regulatory mechanisms of the longissimus dorsi muscle in Longdong black goats at 6 and 12 months of age. The main results are as follows: In terms of muscle morphology, the 6-month-old group showed higher muscle fiber density and smaller fiber diameter, which indicates that this stage is dominated by muscle fiber proliferation. By the 12-month-old stage, muscle fibers had undergone hypertrophic growth, with significant increases in fiber diameter and cross-sectional area; this process was accompanied by a corresponding increase in the proportion of connective tissue. Regarding muscle fiber types, fast-twitch muscle fibers (Type II) were the dominant type in the 6-month-old group. At 12 months of age, the proportion of slow-twitch muscle fibers (Type I) increased, and this change was accompanied by a metabolic shift from glycolysis to oxidative phosphorylation. Transcriptome analysis identified 387 differentially expressed genes (DEGs) in total. Functional enrichment analysis showed that these DEGs were mainly involved in biological processes such as skeletal muscle growth and glucose metabolism and were significantly enriched in pathways including arginine–proline metabolism and the AMPK signaling pathway. Collectively, these pathways drive muscle development by regulating energy homeostasis and the structural maturation of muscle fibers. Based on the above results, it is suggested that during the 6-month-old stage, protein nutrition should be strengthened to promote muscle fiber proliferation; during the 12-month-old stage, energy intake should be rationally regulated to improve meat quality.

## Figures and Tables

**Table 1:** Summary of programs and software tools for data analysis and processing.

Software Name	Version	Purpose in This Study
Image-Pro Plus	6.0	Mainly used for image analysis, including image acquisition and morphological processing
Excel	2016	Preliminary data organization
SPSS	24.0	Used for professional statistical analysis
GraphPad Prism	9	Used for scientific data statistics and high-quality charting

**Table 2:** Information on gene primers.

Gene	Primer Sequence	Primer Length	Annealing Temperature	Sequence Number	Primer Specificity
*HSPB1*	F: CAAGTCAGCTACCCAGTCGG R: TGTTCGGACTTTCCGGCTTC	93	60 °C	XM_018040903.1	Fairly good
*LPL*	F: GAGGCCTTGGAGATGTGGAC R: AATTGCACCGGTACGCCTTA	114	60 °C	NM_001285607.2	
*COL1A1*	F: AAATGGAGCTCCTGGTCAGATG R: AGCACCATCATTTCCTCTAGCAC	100	60 °C	XM_018064895.1	
*TET2*	F: GCCTAACCCACCGACTCTTC R: CTTGCTGTTTGTGCCCCATC	77	60 °C	XM_013964483.2	
*SCD*	F: GTGCCGTGGTATCTATGGGG R: ACAACAGCGTACCGGAGAAG	74	60 °C	NM_001285619.1	
*SLC25A4*	F: AGTTCACTGGTCTGGGCAAC R: TGGACCGAGACGTTGAAACC	88	60 °C	XM_018042040.1	
*PPARA*	F: TTCCCTCTTTGTGGCTGCTA R: GCGTCGTCAGGATGGTTGTT	135	60 °C	XM_018048905.1	
*CAPN2*	F: CATCCGGGTCTTTTCCGAGA R: GATGTCGTCCTCGCTGATGT	97	60 °C	XM_018060202.1	
*GAPDH*	F: AAGGTCGGAGTGAACGGATT R: ACGATGTCCACTTTGCCAGTA	80	60 °C	XM_005680968.3	

**Table 3:** Partial results of KEGG pathways and differentially expressed genes enrichment.

Group	Genes	Signaling Pathways	Expression
B6_vs_B12	*ACACB* *CPT1A*	AMPK signaling pathway	Down
	*ADCY6* *ADCY7*	Gap junction	Down
	*NOS1* *SMOX*	Arginine and proline metabolism	Down

## Data Availability

The data presented in this study are available on request from the corresponding author.

## Supplementary Materials

The following supporting information can be downloaded at https://www.mdpi.com/article/10.3390/ani15223265/s1, Figure S1: Distribution of Bases and Quality Scores. Table S1: Main instruments and related information used in this experiment. Table S2: Specific information of experimental reagents. Table S3: Primary antibodies, secondary antibodies and corresponding antigen retrieval conditions used in this experiment. Table S4: Statistics of sequencing data. Table S5: Information on alignment to goat reference genome. Table S6: Principal Component Analysis.
