# Supplementary material for: Study on the Development and Formation Specifics of Longissimus Dorsi Muscles in Ziwuling Black Goats
Source: Animals (Basel). 2025 Nov 11;15(22):3265. doi: 10.3390/ani15223265 (PMC12649415; doi:10.3390/ani15223265)
Supplement: Supplementary file 1 [file animals-15-03265-s001.zip › Supplementary Tables S1-S6.pdf]

Supplementary Table S1 Main instruments and related information used in this experiment

Name	Manufacturer	Model
Dehydrator	DIPATH (Italy)	Donatello
Embedding Machine	Wuhan Junjie Electronics Co., Ltd.	JB-P5
Pathological Microtome	Shanghai Leica Instruments Co., Ltd.	RM2016
Freezing Stage	Wuhan Junjie Electronics Co., Ltd.	JB-L5
Tissue Flattener	Zhejiang Jinhua Kedi Instrument Equipment Co., Ltd.	KD-P
Decolorizing Shaker	Wuhan Servicebio Technology Co., Ltd.	DS-2S100
Histochemical Pen	Wuhan Servicebio Technology Co., Ltd.	G6100
Upright Fluorescence Microscope	Nikon (Japan)	Nikon Eclipse C1
Scanner	3DHISTECH	Pannoramic MIDI

Supplementary Table S2 Specific information of experimental reagents

Reagent	Manufacturer	Product Code
Anhydrous Ethanol	Sinopharm Chemical Reagent Co., Ltd.	100092183
Environmentally Friendly Dewaxing Solution	Wuhan Servicebio Technology Co., Ltd.	G1128
Citric Acid Antigen Retrieval Solution (pH 6.0)		G1202
EDTA Antigen Retrieval Solution (pH 9.0)		G1203
EDTA Antigen Retrieval Solution (pH 8.0)		G1206
PBS Buffer Solution		G0002
Tissue Autofluorescence Quencher		G1221
Bovine Serum Albumin (BSA)		GC305010
DAPI Staining Reagent		G1012
Anti-Fluorescence Quenching Mounting Medium		G1401

Supplementary Table S3 Primary antibodies, secondary antibodies and corresponding antigen retrieval conditions used in this experiment

Primary Antibody Name	Primary Antibody Product Code	Primary Antibody Manufacturer	Primary Antibody Species	Primary Antibody Dilution Ratio	Corresponding Secondary Antibody Name	Secondary Antibody Product Code	Secondary Antibody Manufacturer	Secondary Antibody Dilution Ratio
fast	GB112589	Servicebio	Rabbit	1:300	CY3-labeled Goat Anti-Rabbit IgG	GB21303	Servicebio	1:300
Slow	GB121857	Servicebio	Mouse	1:500	Alexa Fluor 488-labeled Goat Anti-Mouse IgG	GB25301	Servicebio	1:400

Antigen retrieval conditions: EDTA (pH 8.0) with microwave treatment: medium fire for 10 minutes, fire off for 5 minutes, then medium-low fire for 5 minutes, fire off for 2 minutes, and medium-low fire for 5 minutes.

Supplementary Table S4 Statistics of sequencing data

Samples	Clean reads	Clean bases	GC Content	%≥Q30
B61	21,320,953	6,353,412,931	48.23	94.46
B62	19,478,692	5,794,065,775	49.59	94.96
B62	19,474,791	5,800,727,503	49.04	94.62
B64	19,746,184	5,884,623,592	50.53	94.55
B65	19,478,132	5,810,807,530	49.56	94.56
B66	19,184,760	5,726,060,331	49.61	94.47
B121	19,417,544	5,787,487,676	47.58	94.88
B122	19,300,715	5,750,077,004	49.28	94.42

B123	19,492,566	5,775,754,350	50.98	94.21
B124	19,687,301	5,873,526,302	48.79	94.81
B125	19,230,228	5,739,736,364	49.36	94.65
B126	19,649,318	5,872,695,744	48.83	95.13

Supplementary Table S5 Information on alignment to goat reference genome

Sample	Total Reads1	Mapped Reads2	Uniq Mapped Reads3	Multiple Map Reads4	Reads Map to '+'5	Reads Map to '-'6
B61	42,641,906	29,849,529 (70.00%)	28,187,303 (66.10%)	1,662,226 (3.90%)	16,339,399 (38.32%)	16,310,088 (38.25%)
B62	38,957,384	29,494,655 (75.71%)	27,730,670 (71.18%)	1,763,985 (4.53%)	16,239,069 (41.68%)	16,218,037 (41.63%)
B62	38,949,582	29,321,135 (75.28%)	27,786,862 (71.34%)	1,534,273 (3.94%)	15,852,198 (40.70%)	15,860,902 (40.72%)
B64	39,492,368	31,101,565 (78.75%)	29,303,596 (74.20%)	1,797,969 (4.55%)	16,945,205 (42.91%)	16,934,939 (42.88%)
B65	38,956,264	30,258,404 (77.67%)	28,660,499 (73.57%)	1,597,905 (4.10%)	16,447,017 (42.22%)	16,442,200 (42.21%)
B66	38,369,520	27,629,935 (72.01%)	25,478,274 (66.40%)	2,151,661 (5.61%)	16,046,107 (41.82%)	15,930,815 (41.52%)
B121	38,835,088	25,029,102 (64.45%)	23,753,250 (61.16%)	1,275,852 (3.29%)	13,446,707 (34.63%)	13,472,275 (34.69%)
B122	38,601,430	28,852,931 (74.75%)	27,417,739 (71.03%)	1,435,192 (3.72%)	15,599,428 (40.41%)	15,633,888 (40.50%)

B123	38,985,132	30,560,931 (78.39%)	28,782,823 (73.83%)	1,778,108 (4.56%)	16,755,569 (42.98%)	16,809,520 (43.12%)
B124	39,374,602	30,603,045 (77.72%)	28,867,402 (73.31%)	1,735,643 (4.41%)	16,884,642 (42.88%)	16,828,768 (42.74%)
B125	38,460,456	28,102,769 (73.07%)	26,345,642 (68.50%)	1,757,127 (4.57%)	15,645,068 (40.68%)	15,627,367 (40.63%)
B126	39,298,636	29,285,115 (74.52%)	27,720,651 (70.54%)	1,564,464 (3.98%)	15,923,299 (40.52%)	15,919,959 (40.51%)

Supplementary Table S6 Principal Component Analysis

Grouping	Principle component	Proportion of variance	*P*-vaule
B6	B61	0.17118	0.004
	B62	0.14982	
	B63	0.11405	
	B64	0.10208	
	B65	0.09328	
	B66	0.07339	
B12	B121	0.07171	
	B122	0.06392	
	B123	0.05782	
	B124	0.053	
	B125	0.04977	
	B126	0	
