# Supplementary figures and images for: Study on the Development and Formation Specifics of Longissimus Dorsi Muscles in Ziwuling Black Goats
Source: Animals (Basel). 2025 Nov 11;15(22):3265. doi: 10.3390/ani15223265 (PMC12649415; doi:10.3390/ani15223265)

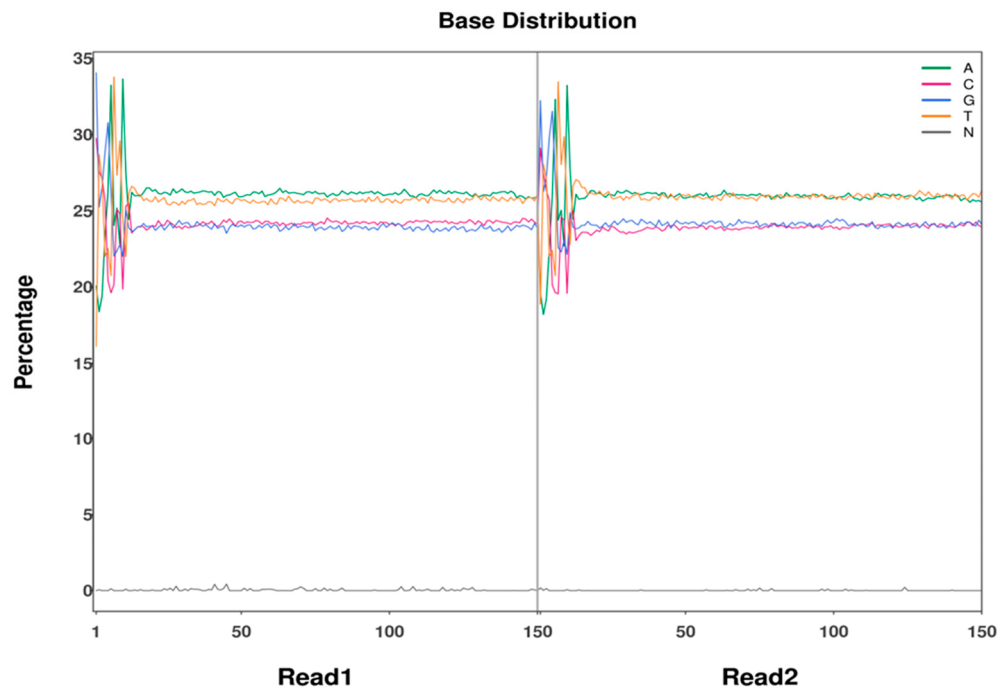

B61

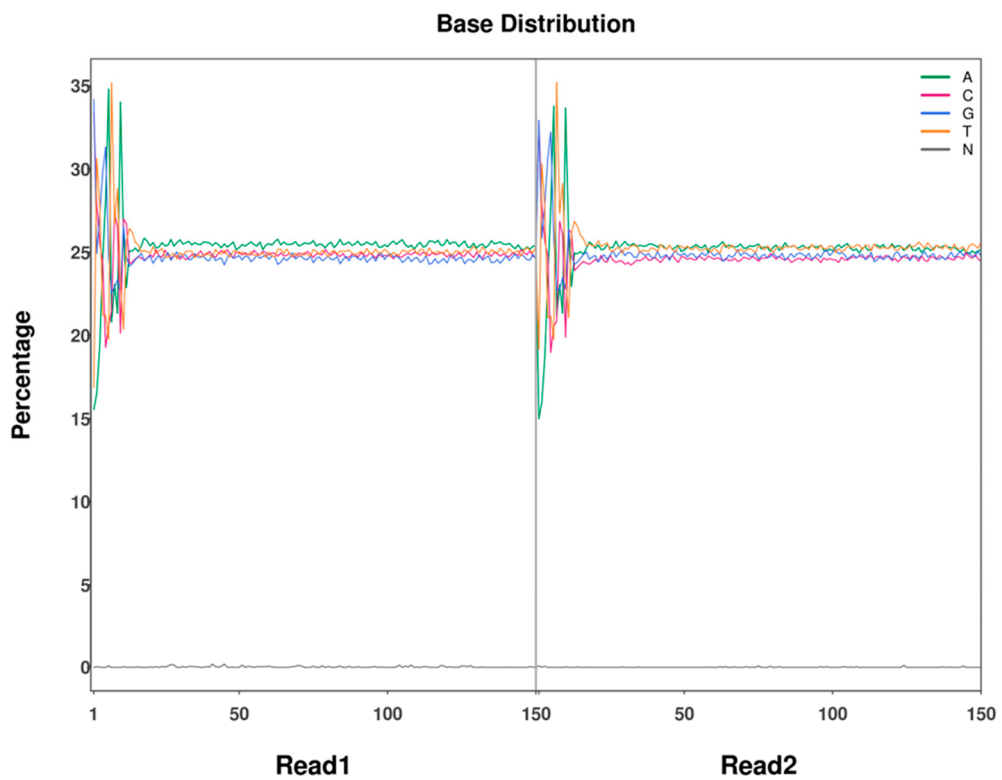

B62

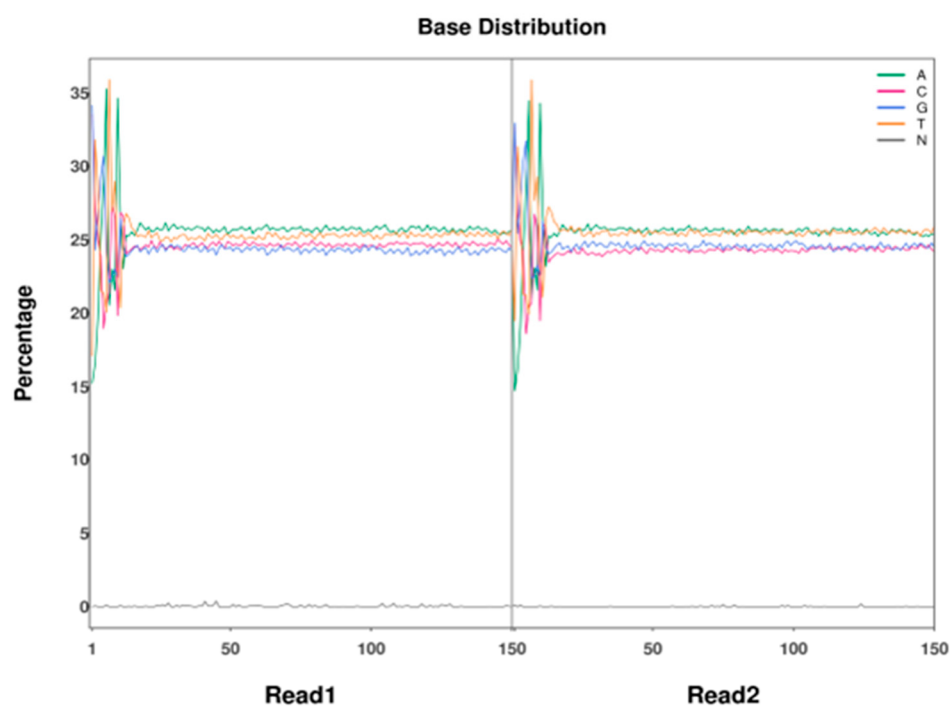

B63

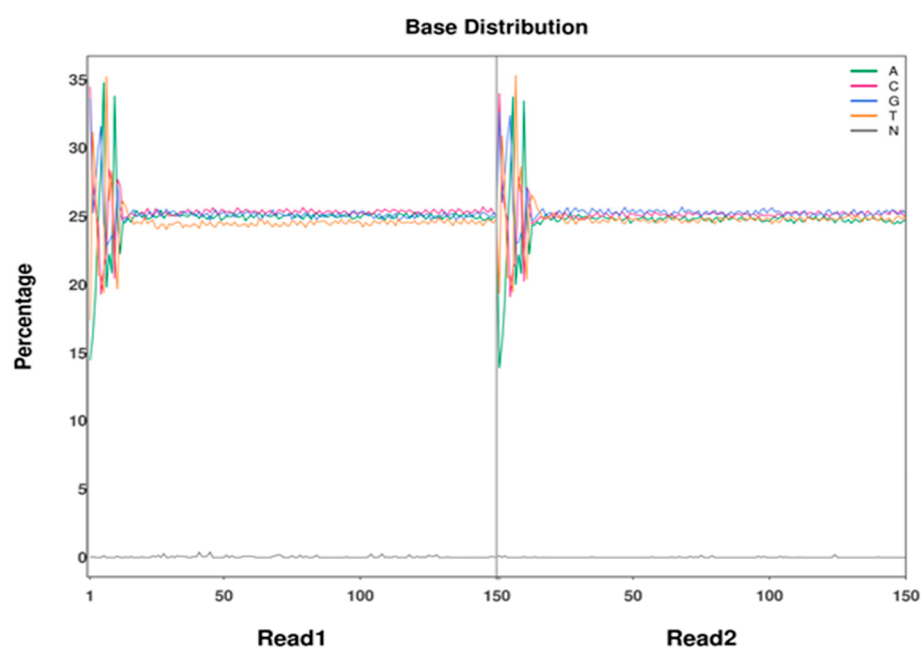

B64

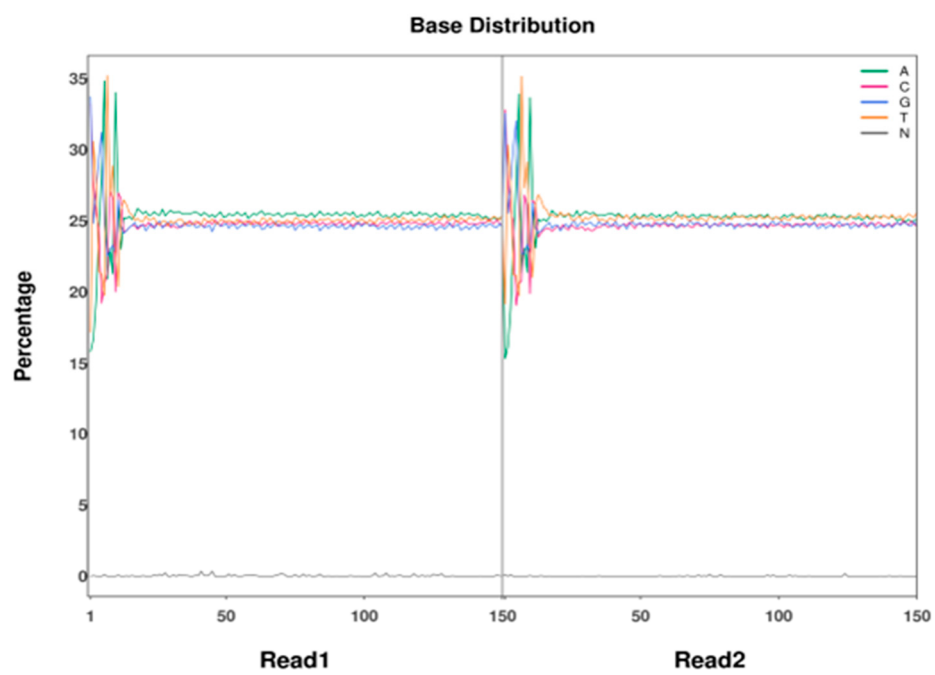

B65

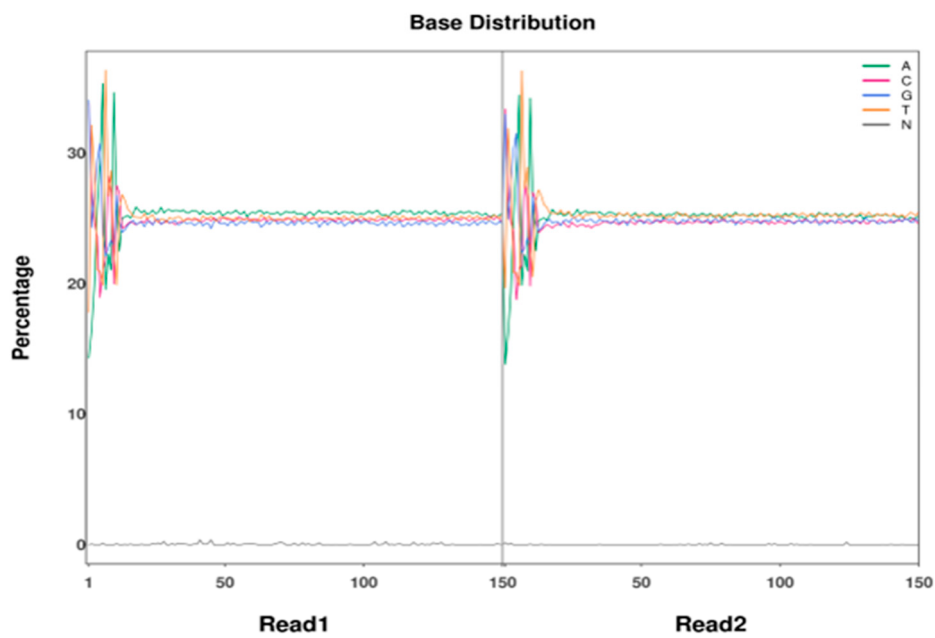

B66

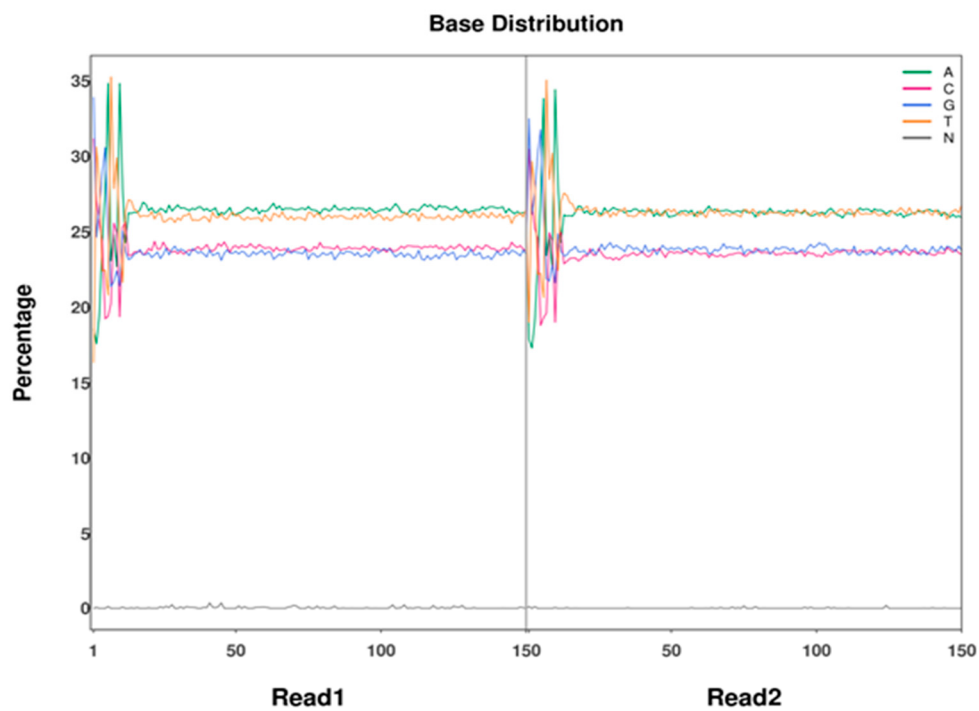

B121

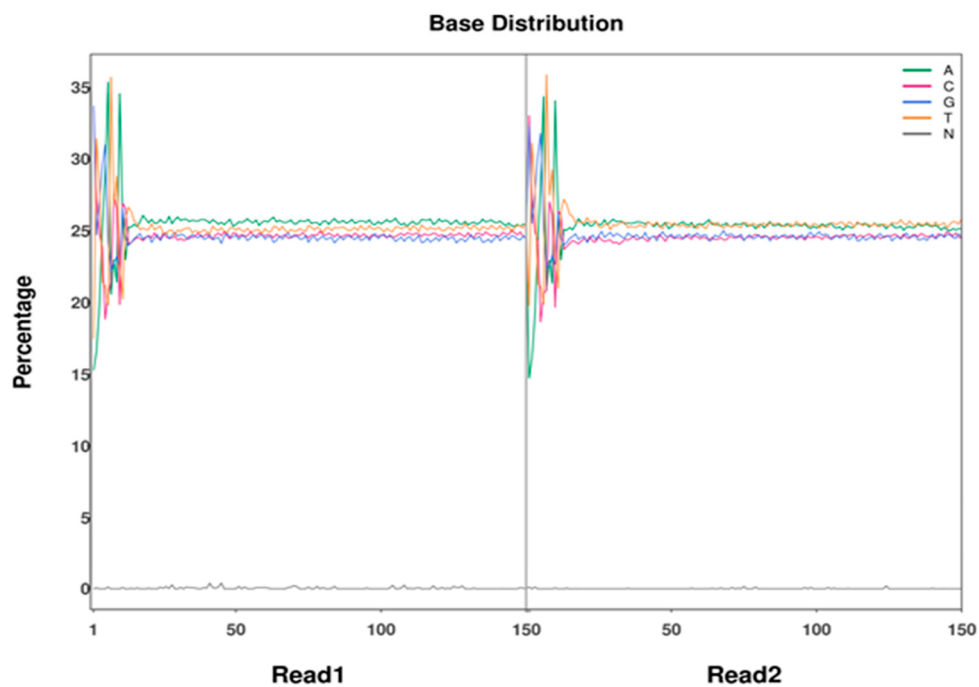

B122

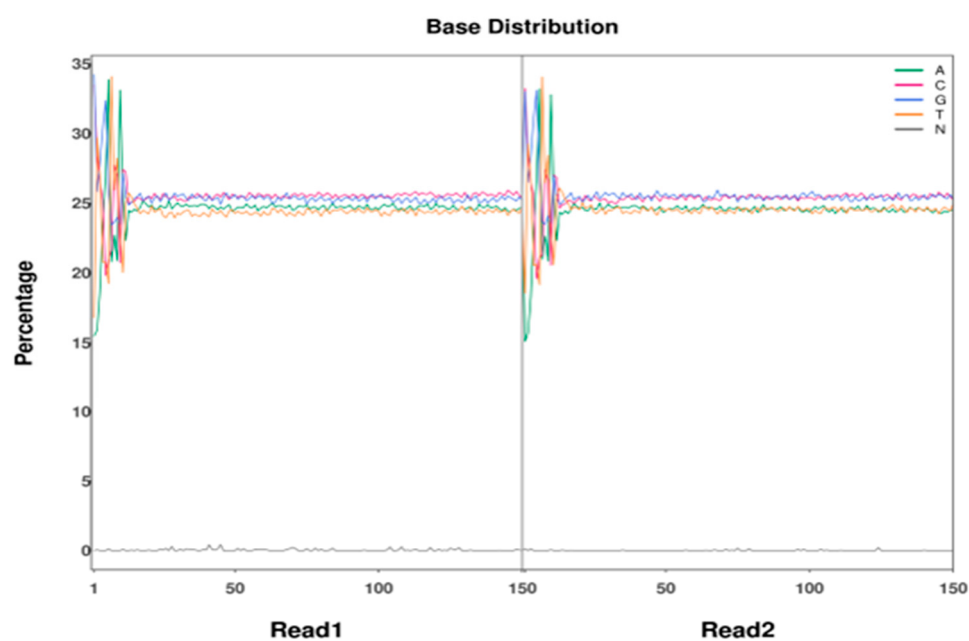

B123

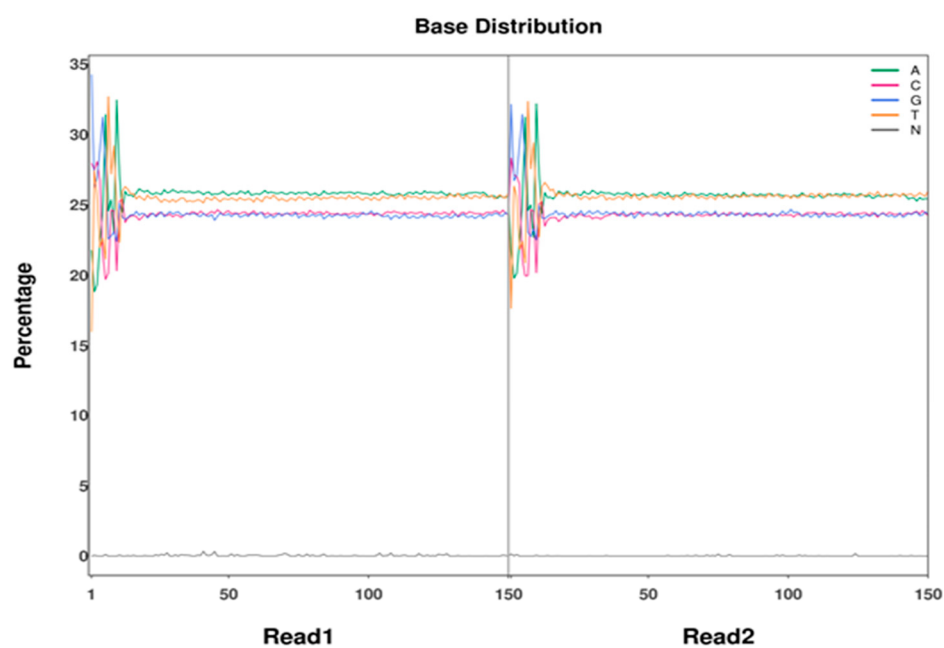

B124

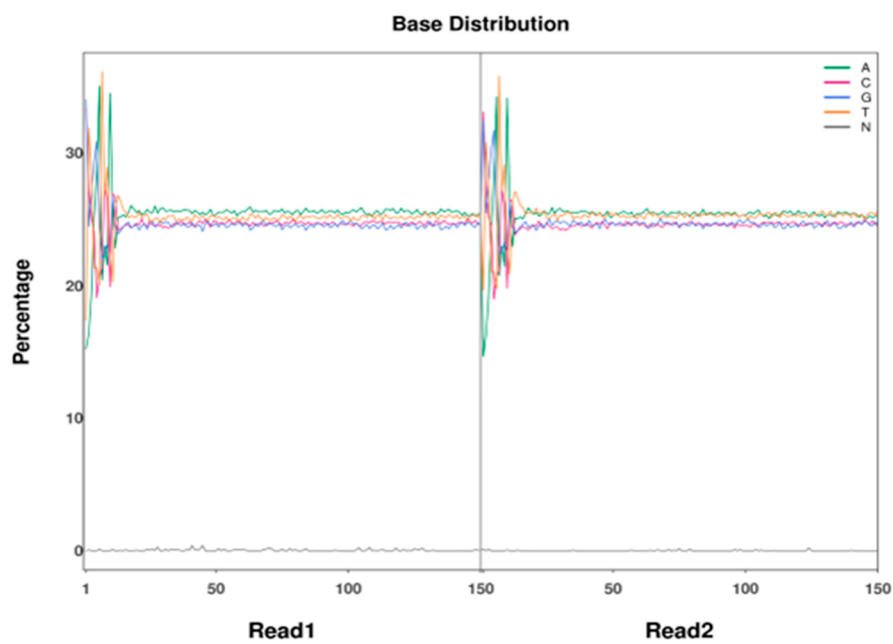

B125

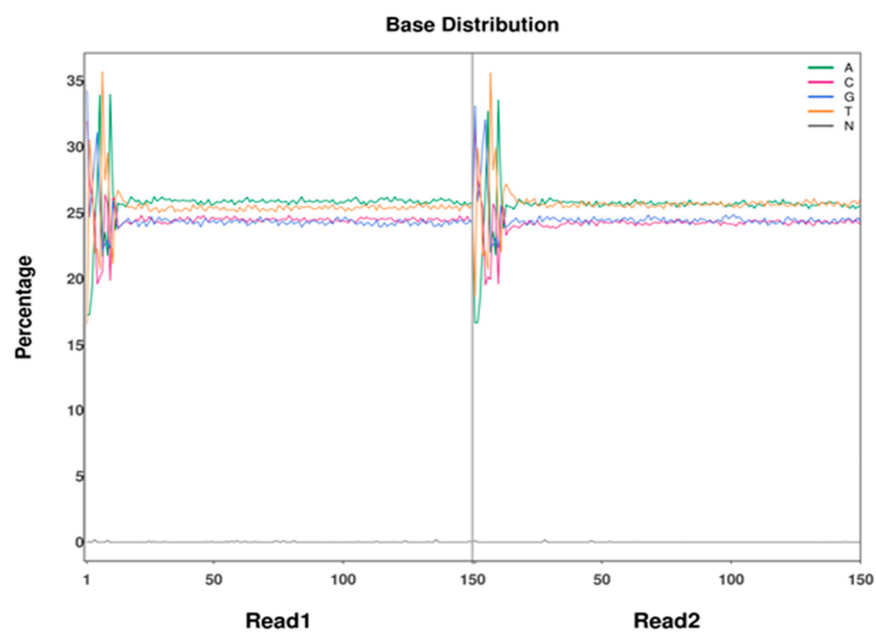

B126

Supplementary Figure S1 Distribution of Bases and Quality Scores

Supplement: Supplementary file 1 [file animals-15-03265-s001.zip › Supplementary Figure S1.pdf]
